# Spinal Cavernous Malformations: A Narrative Review

**DOI:** 10.3390/neurosci7010017

**Published:** 2026-02-02

**Authors:** Aleeza Safdar, Ali Osman, Rouzbeh Motiei-Langroudi

**Affiliations:** 1 Department of Neurosurgery, University of Kentucky, Lexington, KY 40536, USA; 2 Heritage College of Osteopathic Medicine, Ohio University, Dublin, OH 43016, USA

**Keywords:** spinal cord, cavernous malformation, cavernoma, treatment

## Abstract

The management of spinal cord cavernous malformations (SCCMs) involves critical decisions between surgical and conservative treatments, informed by the patient’s preoperative neurological status, lesion characteristics, and timing of intervention (early or delayed surgery). Surgery remains an option for symptomatic patients, especially those with significant or progressive neurological deficits and large lesions, aiming for gross total excision to prevent (re)hemorrhage and improve outcomes. Conversely, conservative management is appropriate for small, asymptomatic lesions, with regular monitoring to detect changes necessitating surgery. Studies highlight the benefits and risks of both approaches. Surgical resection typically leads to neurological recovery, although worse preoperative status and larger lesions predict poorer outcomes. Other factors influencing surgical success include lesion location and timing of surgery, with early surgery (within 3 months) generally yielding better long-term outcomes. Future research should focus on the optimal timing of surgery, particularly the benefits of urgent intervention.

## 1. Introduction

Spinal cord cavernous malformations (SCCMs) or spinal cavernomas are rare, well-circumscribed intramedullary vascular lesions with a mulberry-like appearance. Histologically, they are multi-lobulated, sinusoidal, endothelin-lined spaces that lack tight junctions and do not possess neural parenchyma. SCCMs are histologically identical to cranial cavernous malformations (CCMs), with the exception that calcification is less commonly seen in SCCMs in comparison to their cranial counterparts [1].

### 1.1. Epidemiology

Overall, SCCMs make up about 5 percent of all cavernous malformations of the central nervous system and 5–12% of intraspinal vascular malformations. The prevalence of spinal cavernomas in the general population is not well-documented, primarily due to their rarity and the asymptomatic nature of many cases. The average age at presentation is 39–42 years with an equal male to female distribution. They are mostly located in the thoracic spine, followed by the cervical and cervico-medullary junction. Approximately 40–47% of patients with SCCM also have an intracranial lesion on screening imaging, and nearly 34% of patients have an associated venous malformation; 12% of patients have familial cavernous malformation [2]. Moreover, cases of multiple SCCMs in the absence of widespread cranial involvement have rarely been reported, primarily in small case reports and limited case series [3,4,5].

While familial cavernous malformation syndromes associated with mutations in KRIT1 (CCM1), CCM2, and PDCD10 (CCM3) are well established for cavernous malformations in general and CCMs specifically, no genetic mutations have been shown to specifically predispose to SCCMs [6]. Although SCCMs may occur in the setting of familial disease, the majority of reported cases appear sporadic, and no distinct genetic signature unique to SCCMs has been identified [7].

### 1.2. Diagnosis and Imaging Studies

**Figure 1:**
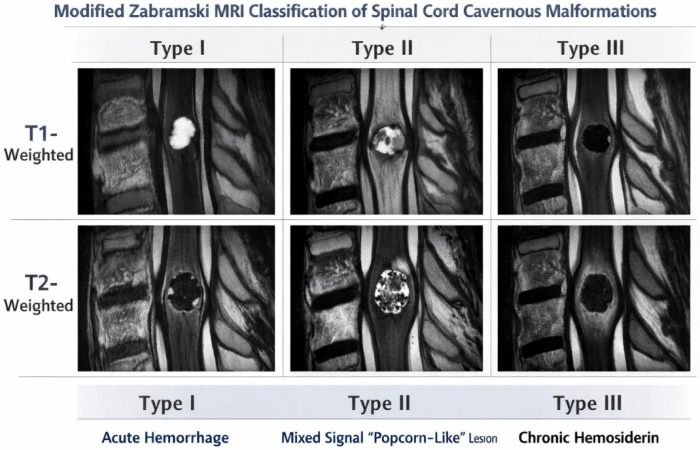
A schematic representation of Modified Zabramski MRI classification for spinal cord cavernous malformations is shown here.

SCCMs are angiographically occult, and diagnosis is made by Magnetic Resonance Imaging (MRI). They present as small (approximately 1 cm in size) intramedullary lesions with a hypointense rim and reticulated core of heterogenous signal intensity on T2-MRI. The Zabramski classification is commonly used to describe the appearance of CCMs based on MRI signal intensity and pathological characteristics [6]. A modified version of the Zabramski classification was used in a recent study to describe SCCM appearance on MRI only [8]. According to the modified classification, Type I lesions will usually be hyperintense on T1-weighted images due to acute to subacute hemorrhage but demonstrate variable intensity on T2-weighted sequences. Type II lesions will demonstrate characteristic mixed-signal intensity appearance on both T1- and T2-weighted images due to hemorrhage of different ages with peripheral hypointense hemosiderin ring. Type III lesions represent chronic lesions that are iso- to hypointense on T1-weighted imaging and hypointense on T2-weighted imaging due to hemosiderin (Table 1 and Figure 1).

## 2. Presentation, Natural Course, and Bleeding Rate in SCCMs

Many studies are available that predict the natural course for CCMs and describe a rather benign course of the disease, with an estimated occurrence of intracerebral hemorrhage of around 20% over 5 years for intracranial cavernous malformations [5,8,9,10,11,12,13]. However, studies investigating the course of SCCMs are rare and limited by a small sample size and short follow-up time due to the rarity of the disease, and they mainly focus on surgical outcomes [14,15,16,17,18,19,20,21,22,23,24,25,26,27,28,29]. Recent multicenter cohort studies and contemporary surgical series have further refined hemorrhage risk stratification, surgical timing considerations, and outcome predictors in SCCMs, reinforcing the importance of individualized management strategies [30]. SCCMs are a major cause of intramedullary hemorrhage [19,31], with a cumulative 5-year hemorrhage risk around 40% and annual rates ranging from 1.4 to 10% (average 2.1%). Symptomatic lesions have higher annual hemorrhage rates (9.5–17.6%) compared to asymptomatic ones (0.8%) [18,19,24,25,31,32,33]. Most natural history studies are limited by a small sample size and short follow-up due to surgical removal or loss to follow-up. In general, SCCMs demonstrate a more aggressive clinical behavior compared to CCMs [18], with higher rates of bleeding and significant morbidity following intramedullary hemorrhage such as severe muscle weakness, paraplegia, or quadriplegia. Studies on the natural course of SCCMs are summarized in Table 2.

A recent multicenter study by Ren et al. reported 305 symptomatic patients with SCCMs, of whom 83% presented with hemorrhage. The overall annual prospective hemorrhage rate was 8.5% per person per year, and the cumulative risk of hemorrhage was 35.1% at 5 years. Patients with a history of prior hemorrhage, Zabramski Type I appearance on MRI, pediatric age, and a worse baseline neurological status had a significantly higher subsequent hemorrhage risk and poorer long-term outcomes. Lesions located in the thoracic cord and those with familial form of the disease also demonstrated worse outcomes. The study, however, was limited by a short follow-up in most patients and the inclusion of only symptomatic cases, which may not represent the course of incidentally detected lesions. The management may also vary for superficial easily accessible lesions compared to smaller deep lesions [8].

These lesions can become symptomatic due to the high density of eloquent spinal cord tissue, mass effect, or new hemorrhage causing pain, myelopathy, or radiculopathy depending on the lesion location and extent of bleeding. Motor weakness is the most commonly presenting symptom (29–61%), followed by pain (52.1%), sensory disturbance (34%), gait ataxia (26.8%), and urinary or bowel dysfunction in 15.5–24%. Less commonly, cervical lesions may cause respiratory distress (0.5%) [31].

In terms of symptom presentation, three main clinical patterns have been described: recurrent stepwise neurological decline with partial recovery, slow progressive deterioration due to microhemorrhages and gliosis, and acute or rapidly worsening symptoms from large hemorrhage or rupture beyond the lesion capsule [1,25,34].

In a ten-year multicenter study that included patients with cerebral and spinal cavernous malformations managed conservatively, only 6 of 91 patients (6.6%) were found to have SCCMs. The cumulative 10-year hemorrhage risk for SCCM was 67%, compared to 10% for all cavernous malformations. The annual hemorrhage risk was 7% for SCCM versus 4% overall and 0% in asymptomatic patients. Although overall risk decreased over time, SCCM patients with initial hemorrhage showed increasing risk and worse outcomes, suggesting a more aggressive course and supporting surgical treatment [35].

An earlier study by the same group followed 71 SCCM patients for a mean of 25.3 ± 41.9 months [31]. Most were symptomatic (92.5%), with 62% presenting with intramedullary hemorrhage and only 8.5% asymptomatic. The 5-year cumulative hemorrhage risk was 41.3%, and those initially presenting with bleeding had a 55.7% risk of rebleeding. Compared to CCMs, SCCMs showed higher rates of symptomatology and hemorrhage (92.5% vs. 33% symptomatic; 62% vs. 25.3% hemorrhagic) [9,29].

In a retrospective series by Goyal et al., 85 SCCM patients were analyzed: 21 (24.7%) underwent early surgery and 64 (75.3%) were managed conservatively. Among conservatively treated patients, 25% had hemorrhage during follow-up and 17% eventually required surgery. The overall annual hemorrhage risk was 5.5% per person per year (9.5% symptomatic vs. 0.8% asymptomatic). Larger lesion size (>1 cm), prior hemorrhage, and symptomatic presentation predicted higher risk [19].

Similarly, in a smaller report of 10 SCCM patients (mean age 34.5 years), all developed neurological deficits after one or more hemorrhages (1–5 per patient). The retrospective annual hemorrhage rate was 4.5%, and the prospective rehemorrhage rate reached 66% per person per year. Postoperatively, four patients improved and six remained stable, leading authors to recommend surgery for symptomatic SCCMs [29].

However, not all studies of natural course have supported this aggressive course for SCCMs. Kharkar et al. retrospectively reviewed 14 patients with a mean age of 42 years with symptomatic SCCMs to describe the clinical presentation and natural history. All patients were symptomatic at presentation. Ten patients (71%) were treated conservatively, and four patients (29%) were treated surgically. The mean time from onset of symptoms to presentation was 10 months. The mean follow-up for the conservatively managed patients was 80 months. No conservatively managed patients experienced new intramedullary hemorrhage during the follow-up period, and 9 out of 10 had the same or improved Modified McCormick Scale score at last follow-up compared with presentation. Among those surgically managed, two remained stable, one improved, and one had worse disease at the last evaluation. The authors, therefore, concluded that among their cohort, it was possible for those with symptomatic SCCMs who were conservatively managed to be clinically stable over long-term follow-up, and thus not all lesions may be progressive [18].

Of note, one major limitation of the literature is the absence of a prospective cohort of purely asymptomatic or randomly found SCCM lesions, to be able to confer natural course or prognostic data for asymptomatic lesions.

## 3. Treatment Options and Management Considerations

### 3.1. Surgical vs. Conservative Treatment, Surgical Considerations, and Outcome of Surgery

Based on available data, microsurgical resection remains the only definitive treatment for SCCMs, particularly in symptomatic patients with significant or progressive neurological deficits. In these cases, gross total excision should be attempted. The utility of alternative approaches including focused radiation and radiosurgery in the treatment of symptomatic SCCMs remains unknown. Current evidence does not support routine use of radiosurgery for SCCMs, and it is generally considered a non-standard/experimental approach except possibly in highly selective cases where surgery is not feasible. Asymptomatic patients or those with mild or spontaneously resolving symptoms may be followed expectantly, though surgery should be considered if the lesion is exophytic [1,36,37]. In asymptomatic patients, conservative management typically consists of clinical observation with periodic MRI surveillance to monitor for interval hemorrhage or neurological change. Although standardized imaging follow-up intervals have not been established, regular clinical and radiographic assessment is emphasized across natural history studies [38].

Nagoshi et al. reviewed 66 patients, of whom 57 underwent surgery and 9 were treated conservatively. Preoperative patients who had unstable gait had a significantly higher rate of hemorrhagic episodes as compared to patients with stable gait, as determined by MMS score (52.4% vs. 19.4%, *p* = 0.01). Patients who received conservative treatment had significantly smaller lesions in size (2.5 ± 1.5 mm vs. 5.9 ± 4.1 mm, *p* = 0.02). The data showed that surgery led to significant neurological recovery; however, larger lesion size (8.6 ± 4.5 mm vs. 3.5 ± 1.6 mm, *p* = 0.01) and worse preoperative neurological status were predictors of a poor outcome. The authors recommended evacuation of a hemorrhage upon its occurrence to prevent (re)hemorrhage and deterioration. Smaller lesions can be followed conservatively with periodic MRI evaluation [39].

A case series of 96 patients (81 surgical, 15 conservative) reported a mean symptom duration of 19.7 months and long-term follow-up (mean 45.8 months) in 75 patients (64 surgical, 11 conservative). Most patients experienced gradual neurological decline before intervention. At final follow-up, 36% of surgical patients improved, 55% were unchanged, and 9% worsened. Complete resection was achieved in 60 cases. Among conservative patients, 45% improved and 55% remained stable, with no deterioration. The authors concluded that complete microsurgical resection of symptomatic SCCMs is safe and prevents rebleeding, while smaller or ventrally located lesions may be managed conservatively to avoid surgical risk [17].

In another retrospective series of 85 patients (mean age 40.5 years, mean follow-up 42.8 months), 58 underwent microsurgical removal and 27 were conservatively managed. In the surgical group, 69.0% of patients improved, 27.6% were stable, and 3.4% worsened. In the conservatively managed group, 14.8% of patients improved, 70.4% were stable, and 14.8% worsened. After adjustment for lesion size and location, no difference was found in long-term neurological status. The annual risk of hemorrhage in conservatively managed patients was 3.9%. No patient experienced rebleeding after microsurgical resection. These data suggest that surgical resection ablates the risk for recurrent hemorrhage and allows for good outcome in well-selected patients [28]. Another report of 25 patients (20 surgical, 5 conservative) showed that 90% of surgically treated patients were Frankel D and 10% Frankel C at baseline. None worsened at discharge; 80% remained unchanged and 20% improved during early follow-up (mean 6.3 months). All improved cases had superficially located lesions and underwent early surgery (≤3 months). No deterioration occurred over long-term follow-up (mean 44.7 months). Conservatively managed patients remained neurologically stable for an average of 6.7 years. The authors concluded that SCCMs can be resected safely with favorable outcomes, though conservatively managed patients may remain stable [14], again emphasizing proper patient selection. A retrospective analysis of 48 patients with SCCMs over 28 years showed that factors predictive of unfavorable outcome were thoracolumbar level lesions and poorer preoperative function as graded with the Epstein–Cooper and ASIA scale (A–C) and preoperative neurological status was the main determinant of outcome [16].

In those treated surgically, a posterior approach is the standard, but extensive modification of the approach is necessary for ventral or more anterior lesions. For instance, sectioning of the dentate ligament, micro-rotation of the spinal cord, and a tailored myelotomy can allow exposure of lateral or ventral lesions but must be performed carefully. In a study on 22 patients, dorsal lesions (mean lesion diameter 1.0 ± 0.4 cm) were resected with laminectomy, while ventral and lateral lesions were approached with unilateral radical facetectomy and resection of the dentate ligament. The mean operative time was 4.0 ± 1.0 h, and there was one case of wound infection (5% complication rate). Neurological status was improved (mean MMS score improved from 1.8 ± 1.2 preoperatively to 1.3 ± 0.7 at last follow-up). At long-term follow-up, 50% of patients were stable, 41% improved, and 9% had worse neurological status related to the development of dysesthetic pain. This occurred most commonly in patients with a long preoperative history of symptoms [40]. Other authors have suggested that a less aggressive approach is better for ventral lesions [17]. Transient postoperative deficits can occur but often improve in the postoperative course. In this series with the posterior approach, 41% improved, 50% were stable, and 9% were worse, usually secondary to postoperative dysesthesia. Complications that required reoperation occurred in 5% of cases, and complete resection was radiographically confirmed in all patients [40]. In another multicenter retrospective cohort of 53 patients with SCCMs, 37 were treated surgically. Posteriorly placed lesions had better outcomes than anterior lesions. At the last follow-up, 22 had MMS grades of 1–2 (independent), 12 had grades of 3 (requiring assistance), and 3 had grades of 4 (bedridden). Surgery led to overall neurological improvement in over half of the patients [17]. In another series of 80 patients with SCCMs who underwent surgical resection, 11% deteriorated, 83% were unchanged, and 6% improved immediately after surgery compared with their preoperative Frankel grade. At a mean follow-up of five years, 10% of patients were worse, 68% were unchanged, and 23% were improved. Residual or recurrent symptomatic lesions required reoperation in 5% of patients. Early postoperative complications (CSF leakage and deep venous thrombosis) occurred in 6% and late postoperative problems (kyphosis, stenosis, and cord tethering) in 14%. Outcome was found to be significantly associated with the anteroposterior dimension of the lesion [21]. In another review of 83 patients who underwent resection of an SCCM, 63 patients had long-term follow-up. Among these, 19 improved, 39 were stable, and 5 declined. Patients who had surgery within three months of the onset of symptoms were more likely to have a neurological recovery. This finding suggests that early resection can prevent sequelae that result from hemorrhage [23]. In a similar review of 98 SCCMs, 42% of patients improved, 51% remained stable, and 7% worsened. Dorsal or superficial lesions were associated with better outcomes than ventral or more deeply located lesions. Patients who underwent surgery within three months of the onset of their symptoms were more likely to achieve neurological recovery. These data support early microsurgical resection (within 3 months of symptom development), when complete resection is possible, as providing the best opportunity for neurological improvement and prevention of recurrent hemorrhage, while ventral or deep lateral lesions may require a more conservative surgical approach [41]. Studies reporting outcomes of surgery for SCCMs are summarized in Table 3. Studies comparing surgical vs. conservative management outcomes are summarized in Table 4.

### 3.2. Timing of Surgery

While there is a general consensus on indication for surgery of SCCMs, the optimal timing of surgery is less clear [39]. It has been reported that timing of surgery can influence the long-term outcomes in patients with SCCMs [42,44,49,50]. Studies focusing on timing of surgery SCCMs are summarized in Table 5.

In a recent retrospective study of 279 surgically treated patients followed for at least 6 months, it has been reported that patients with a severe baseline neurological impairment represented by an MMS score between 3 and 5 benefitted significantly from early surgery (within 6 weeks), which resulted in an improvement in the mean MMS score of 2.6 ± 1.2 at admission to 2.0 ± 1.0 at last follow-up. They separately analyzed a group of 69 patients who experienced a single severe hemorrhage and found that 52 patients (75.3%) had an improved outcome at last follow-up. Among this subgroup as well, patients who underwent early surgery (<6 weeks) had a significant influence on improvement of MMS score at final follow-up compared to those who underwent surgery after 6 weeks. In patients with severe neurological impairment such as paraplegia or quadriplegia (MMS score = 5), performing surgery during the hyperacute phase (<2 weeks) had an advantage compared to those who underwent surgery later in the acute stage (2–6 weeks) (90% vs. 83% improved, respectively). This, however, did not reach statistical significance, probably due to a limited sample size [50]. The role and benefit of emergent or urgent intervention (within hours or few days after the advent of a severe neurologic deficit or bleeding) are not studied at all and should be the focus of future studies.

## 4. Conclusions

The management of SCCMs involves careful consideration of the patient’s neurological status, lesion characteristics, and timing of intervention. Surgical resection remains the mainstay for symptomatic patients to prevent (re)hemorrhage and stabilize or improve neurological function. Conservative management may be appropriate for small, asymptomatic lesions with regular monitoring to detect any changes that warrant surgical intervention.

## Figures and Tables

**Table 1:** Modified Zabramski MRI classification for spinal cord cavernous malformations.

	T1-Weighted Image	T2-Weighted Image
**Type I**	Hyper	Hyper or hypo
**Type II**	Mixed (hyper and hypo)	Mixed (hyper and hypo)
**Type III**	Iso to hypo	Hypo

**Table 2:** Studies on the natural course of spinal cord cavernous malformations.

Author(s)	Year	Title	Journal	Annual Hemorrhage Rate	Symptomatic Rate	Number of Patients	Improved	Same	Worse	Prognosticators of Worse Outcome	Scale Used to Assess Neurological Function	Reference Number
Ren et al.	2022	Natural History of Spinal Cord Cavernous Malformations	*Neurosurgery*.	8.5%	100%	305	Not reported	Not reported	Not reported	Prior hemorrhage, pediatric patients, familial form, subsequent hemorrhage, worse baseline neurological status	MMS	[8]
Santos et al.	2022	Natural course of untreated spinal cord cavernous malformations	*J Neurosurg Spine*.	10%	91.5%	71	Not reported	Not reported	Not reported	Prior hemorrhage	MMS	[31]
Liang et al.	2011	Management and prognosis of symptomatic patients with intramedullary spinal cord cavernoma	*J Neurosurg Spine.*	Not reported	Not reported	11	5	6	0		Aminoff–Logue	[17]
Kharkar et al.	2007	The natural history of conservatively managed symptomatic intramedullary spinal cord cavernomas	*Neurosurgery*.	Not reported	100%	10	9	0	1		MMS	[18]

**Table 3:** Studies reporting outcome of surgery in spinal cord cavernous malformations.

Author	Year	Title	Journal	Number of Patients	Improved	Same	Worse	Significant Predictors of Better Outcome	Significant Predictors of Worse Outcome	Scale Used to Assess Neurological Status	Reference Number
Ardeshiri et al.	2016	A retrospective and consecutive analysis of the epidemiology and management of spinal cavernomas over the last 20 years in a single center	*Neurosurg Rev*.	20	4	16	0	Superficial location, early surgery (<3 months)	-	Frankel	[14]
Ohnishi et al.	2020	Conservative and surgical management of spinal cord cavernous malformations	*World Neurosurg X*.	13	11	2	0	-	-	MMS	[15]
Reitz et al.	2015	Intramedullary spinal cavernoma: clinical presentation, microsurgical approach, and long-term outcome in a cohort of 48 patients	*Neurosurg Focus*.	48	11	34	3	-	Thoracolumbar localization, worse Preoperative neurological status	ASIA, Epstein and Cooper	[16]
Liang et al.	2011	Management and prognosis of symptomatic patients with intramedullary spinal cord cavernoma	*J Neurosurg Spine*.	64	23	35	6	-	Small lesion, ventral location	Aminoff–Logue	[17]
Kharkar et al.	2007	The natural history of conservatively managed symptomatic intramedullary spinal cord cavernomas	*Neurosurgery*.	4	1	2	1	-	-	MMS	[18]
Goyal et al.	2019	Clinical presentation, natural history and outcomes of intramedullary spinal cord cavernous malformations	*J Neurol Neurosurg Psychiatry*.	32	-	-	-	-	Large, symptomatic, prior hemorrhage		[19]
Labauge et al.	2008	Outcome in 53 patients with spinal cord cavernomas	*Surg Neurol*.	37	20	6	11	Posterior location	Anterior location	MMS	[20]
Mitha et al.	2011	Outcomes following resection of intramedullary spinal cord cavernous malformations	*J Neurosurg Spine*.	80	5	66	9	Anteroposterior diameter	-	Frankel	[21]
Liao et al.	2022	Surgical outcomes of spinal cavernous malformations	*Front Surg*.	98	41	50	7	Dorsal or superficial lesion, symptoms < 3 months	Ventral or lateral deep lesions, symptoms > 3 months	ASIA	[41]
Lu and Lawton	2010	Clinical presentation and surgical management of intramedullary spinal cord cavernous malformations	*Neurosurg Focus.*	22	9	11	2	-	Presence of dysesthesia and longer duration of symptoms	MMS	[40]
Maslehaty et al.	2011	Symptomatic spinal cavernous malformations: indication for microsurgical treatment and outcome	*Eur Spine J.*	14	7	0	7	-	-	Frankel, MMS	[42]
McCormik et al.	1988	Cavernous malformations of the spinal cord	*Neurosurgery.*	6	5	1	0	-	-	-	[43]
Cai et al.	2023	Surgical outcomes of symptomatic intramedullary spinal cord cavernous malformations	*Neurospine*.	29	19	4	6	Lower preoperative MMS, prolonged course, chronic onset of disease	Acute onset, smaller lesion, higher MMS on presentation, recurrent hemorrhage	MMS	[44]
Azad et al.	2018	Long-term effectiveness of gross-total resection for symptomatic spinal cord cavernous malformations	*Neurosurgery*.	32	7	23	1	Preoperative and improved immediate postoperative Frankel grade	-	Frankel, Aminoff–Logue	[27]
Sandalciog lu et al.	2003	Intramedullary spinal cord cavernous malformations	*Neurosurg Rev*.	10	4	6	0	-	-	Frankel	[29]
Li et al.	2018	Surgical outcomes of spinal cord intramedullary cavernous malformation	*World Neurosurg*.	63	19	39	5	Duration of symptoms < 3 months	Duration of symptoms > 3 months	MMS	[26]
Gembruch et al.	2016	Lumbar extradural, intra- and extraforaminal cavernoma causing lumbar pain	*Open Jounal of Clinical and Medical Case Reports.*	-	-	-	-	-	-	-	[22]
Nagoshi et al.	2019	Clinical outcomes and prognostic factors for cavernous hemangiomas of the spinal cord	*J Neurosurg Spine.*	57	-	-	-	-	Larger lesion, unstable gait	MMS, ASIA	[39]
Kurokawa et al.	2023	Acceptance of early surgery for treatment of spinal cord cavernous malformation in contemporary Japan	*Neurospine*.	160	-	-	18	-	-	MMS	[34]
Duan et al.	2022	The long-term outcome in a cohort of 52 patients with symptomatic intramedullary spinal cavernous hemangioma after microsurgery and emergency rescue surgery	*Front Med (Lausanne)*.	52	25	25	2	Emergency rescue surgery (ERS) for deteriorative patients	Ventral lesion, longer course of disease, lesion at lumbosacral segment	Modified Aminoff–Logue	[45]
Jallo et al.	2006	Clinical presentation and optimal management for intramedullary cavernous malformations	*Neurosurg Focus*.	26	-	-	-	-	-	-	[46]
Deutsch et al.	2000	Spinal intramedullary cavernoma: clinical presentation and surgical outcome	*J Neurosurg*.	16	-	-	-	-	-	-	[47]
Ren et al.	2019	Surgical approaches and long-term outcomes of intramedullary spinal cord cavernous malformations	* J Neurosurg Spine. *	219	189	-	23	Preoperative mild neurological and disability status, cervically located lesions	Mild preoperative function and thoracolumbar-level lesions, moderate-depth lesions (embedded lesions)	ASIA	[48]

**Table 4:** Studies comparing surgical and conservative management for spinal cord cavernomas.

Author (s)	Year	Title	Journal	Annual Hemorrhage Rate	Symptomatic Rate	No of Patients	Surgical	Conservative	Prognosticators of Worse Outcome	Scale Used to Assess Neurological Function	Reference Number
Kharkar et al.	2007	The natural history of conservatively managed symptomatic intramedullary spinal cord cavernomas	*Neurosurgery*.	Not reported	100%	14	4	10	Not reported	MMS	[18]
Goyal et al.	2019	Clinical presentation, natural history and outcomes of intramedullary spinal cord cavernous	*J Neurol Neurosurg Psychiatry*.	5.5%	-	85	32	53	Presence of symptoms Larger size > 1 cm Prior hemorrhage	-	[19]
Sandalcioglu et al.	2003	Intramedullary spinal cord cavernous malformations	*Neurosurg Rev*.	4.5%	100%	10	10	0	-	Frankel	[29]
Zhang et al.	2016	Comparison of outcome between surgical and conservative management of symptomatic spinal cord cavernous malformations	*Neurosurgery*.	3.9%	100%	-	-	27	-	MMS, KPS	[28]
Badhiwala et al.	2014	Surgical outcomes and natural history of intramedullary spinal cord cavernous malformations	*J Neurosurg Spine*.	2.1%	-	-	-	47	Symptom duration > 5 years	-	[24]
Santos et al.	2023	Natural course of cerebral and spinal cavernous malformations	*Sci Rep*.	6.7%	-	6	-	-	Prior hemorrhage Spinal cord localization	-	[35]
Ardeshiri et al.	2016	A retrospective and consecutive analysis of the epidemiology and management of spinal cavernomas over the last 20 years in a single center.	*Neurosurg Rev*.	-	-	25	20	5	-	Frankel	[14]
Ohnishi et al.	2020	Conservative and surgical management of spinal cord cavernous malformations	*World Neurosurg X*.	3.7%	-	18	13	5	-	MMS	[15]
Liang et al.	2011	Management and prognosis of symptomatic patients with intramedullary spinal cord cavernoma	*J Neurosurg Spine*.	-	-	96	81	15	-	Aminoff–Logue	[17]
Labauge et al.	2008	Outcome in 53 patients with spinal cord cavernomas	*Surg Neurol*.	-	98%	53	40	13	Anterior location	MMS	[20]
Nagoshi et al.	2019	Clinical outcomes and prognostic factors for cavernous hemangiomas of the spinal cord	*J Neurosurg Spine.*	-	-	66	57	9	Unstable gait prior to surgery, worse preoperative neurological surgery, large lesion size	MMS, ASIA, JOA	[39]

**Table 5:** Timing of surgery for spinal cord cavernous malformations.

Studies	Year	Title	Journal	No. of Patients	Timing of Surgery	Neurological Status	Worse Outcomes	Prognosticators of Outcome	Reference Number
Imagama et al.	2017	Optimal timing of surgery for intramedullary cavernous hemangioma of the Spinal cord in relation to preoperative motor paresis, disease duration, and tumor volume and location	*Global Spine J.*	41	Early surgery in asymptomatic patients with thoracic tumors and large tumor size	N (no preoperative paresis), CR (complete preoperative motor recovery), NCR (no complete recovery)	Disease duration from onset thoracic location, tumor volume	Complete preoperative motor recovery, stable gait	[51]
Kurokawa et al.	2023	Acceptance of early surgery for treatment of spinal cord cavernous malformation in contemporary Japan	*Neurospine.*	160	>7 days after presentation	MMS	Surgery after 3 months of onset of symptoms for patients with MMS score/grade V, partial or subtotal resection	Presentation to the hospital for surgery, symptom onset to surgery	[34]

## Data Availability

Data sharing is not applicable to this article, as no new data were created or analyzed in this study.
